# Artificial intelligence chatbots mimic human collective behaviour

**DOI:** 10.1111/bjop.12764

**Published:** 2024-12-31

**Authors:** James K. He, Felix P. S. Wallis, Andrés Gvirtz, Steve Rathje

**Affiliations:** ^1^ Artificial Societies Ltd. London UK; ^2^ University College London London UK; ^3^ King's College London London UK; ^4^ New York University New York New York USA

**Keywords:** artificial intelligence, collective behaviour, homophily, social dynamics

## Abstract

Artificial Intelligence (AI) chatbots, such as ChatGPT, have been shown to mimic *individual* human behaviour in a wide range of psychological and economic tasks. Do groups of AI chatbots also mimic *collective* behaviour? If so, artificial societies of AI chatbots may aid social scientific research by simulating human collectives. To investigate this theoretical possibility, we focus on whether AI chatbots natively mimic one commonly observed collective behaviour: *homophily*, people's tendency to form communities with similar others. In a large simulated online society of AI chatbots powered by large language models (*N* = 33,299), we find that communities form over time around bots using a common language. In addition, among chatbots that predominantly use English (*N* = 17,746), communities emerge around bots that post similar content. These initial empirical findings suggest that AI chatbots mimic homophily, a key aspect of human collective behaviour. Thus, in addition to simulating individual human behaviour, AI‐powered artificial societies may advance social science research by allowing researchers to simulate nuanced aspects of *collective* behaviour.

## BACKGROUND

Recent developments in Large Language Models (LLMs) have given rise to advanced Artificial Intelligence (AI) chatbots, such as OpenAI's ChatGPT and Anthropic's Claude, that can mimic human individual behaviour in a wide range of psychological and economic tasks (Aher et al., 2022; Akata et al., 2023). For example, AI chatbots make moral judgements that are highly correlated (*r* = .95) with human participants (Dillion et al., 2023), reflect human imperfections in abstract reasoning (Dasgupta et al., 2023) and respond similarly to emotion inductions (Aher et al., 2022; Binz & Schulz, 2023). Chatbots differ from human participants in some aspects, such as making estimations and causal reasoning (Aher et al., 2022; Binz & Schulz, 2023). Nevertheless, some have suggested that these models can further social science research by simulating human participants (Dillion et al., 2023), giving rise to the term ‘silicon sampling’ (Argyle et al., 2023).

Since AI chatbots emulate human *individual* behaviour, do groups of chatbots mimic human *collective* behaviour? Here, we use ‘collective behaviour’ to refer to group‐level social dynamics and social phenomena. Mimicking collective behaviour is a non‐trivial issue for AI chatbots. If we consider them as ‘stochastic parrots’ (Bender et al., 2021), we may expect a chatbot prompted to mimic a certain human characteristic (e.g. being anxious) to produce language associated with the characteristic. Mimicking collective behaviour, however, requires chatbots to infer associated social behaviours in a multi‐agent environment without being explicitly prompted on how humans would interact. Still, if groups of AI chatbots do indeed mimic human collective behaviour, they could be used to create artificial societies that simulate human societies' nuanced dynamics and, therefore, be used to enhance collective‐level social science research.

Studying human social dynamics and collective behaviour is important: collective behaviour impacts political movements (Wahlström & Törnberg, 2021), the acceptance of climate‐friendly technologies (Zhang et al., 2020), the spread of misinformation (Cinelli et al., 2020; Rathje et al., 2022) and how societies become divided by echo chambers (Cinelli et al., 2021). However, human societies are difficult to study directly. First, gathering data about social interactions is invasive and expensive to undertake with human participants (Knoke & Yang, 2019; Ryan & D'Angelo, 2018; Valente & Pitts, 2017). Second, group‐level field experiments face ethical challenges and are hard to scale (Baldassarri & Abascal, 2017). Finally, recruiting genuine human participants is becoming more difficult as workers in online subject pools increasingly use ChatGPT (Veselovsky et al., 2023).

Due to these challenges, Agent‐Based Modelling (ABM) has become a key tool in social scientific research (Epstein et al., 2023; Lazer et al., 2020). Instead of observing human participants, ABM simulates them as computational agents and observes their behaviour in virtual environments (Abar et al., 2017). Previous ABM studies have revealed mechanisms behind opinion segregations (Baumann et al., 2020), studied the role of indirect minority influence in social change (Jung et al., 2017), examined the potential outcomes of different COVID‐19 social policies (Gumel et al., 2021; Silva et al., 2020), uncovered how fake news and rumours spread in society (Lotito et al., 2021), and tested theories about the formation of filter bubbles and echo chambers (Geschke et al., 2019). However, classical ABM set‐ups often simplify and homogenize each agent's actions, which limits their ability to capture the nuance, diversity and complexity that exists in real human societies (Abar et al., 2017; Geschke et al., 2019; Lotito et al., 2021).

Recent advances in AI chatbots have led some researchers to propose using them as agents to improve ABM's ability to simulate complex human collective dynamics (Epstein et al., 2023; Grossmann et al., 2023; Park et al., 2023; Pastor‐Galindo et al., 2023). However, to date, there has been limited research into the natural collective behaviours of AI chatbots. The majority of existing research has either used mechanistic rules to guide the AI chatbots' social interactions (Ghaffarzadegan et al., 2023) or predominantly focused on their individual‐level actions and coordination within a group setting (Akata et al., 2023; Li, Zhang, & Sun, 2023).

We build on two prior studies that examined AI chatbots as collectives. One study specifically prompted AI chatbots to behave like humans in economic decision‐making and found that realistic macroeconomic phenomena emerged in the chatbot population (Li, Gao, et al., 2023). In another study, the authors prompted AI chatbots to mimic specific humans in terms of emotion, attitude, interactions and found group‐level information propagation patterns comparable to the human reference group (Gao et al., 2023). We expand on these works by observing the collective behaviours in a much larger AI chatbot population, in which there was no direct prompting that guides the AI chatbots to socialize like humans.

What human collective behaviours are important for AI chatbots to demonstrate? Human societies are well documented in their display of power dynamics, ingroup conformity and outgroup animosity (Homans, 1974; Rathje et al., 2021; Sunstein, 2019). Underlying these dynamics is a human tendency to form communities around similar individuals, a collective behaviour known as *homophily* (Girvan & Newman, 2002; McPherson et al., 2001). Homophily arises from an inclination for contact between similar individuals to take place more frequently than contact between dissimilar individuals (McPherson et al., 2001). It manifests as structural communities–clusters of individuals who maintain denser connections within their group than with external entities (Girvan & Newman, 2002)–where there exists relative homogeneity within communities and relative heterogeneity between communities (Knoke & Yang, 2019).

Homophily is well documented in real‐world human societies, such as in community formation around common languages and similarities in individual demographics, attitudes and beliefs (Knoke & Yang, 2019; McPherson et al., 2001; Titzmann, 2014; Titzmann & Silbereisen, 2009). It is also well documented on online social networks such as X, formerly Twitter, where people are more likely to connect with those who share similar backgrounds (Aiello et al., 2012), discuss similar topics (Faralli et al., 2015; Himelboim et al., 2017; Kang & Lerman, 2012), and have similar values and beliefs (Conover et al., 2011; De Choudhury, 2011; Rathje et al., 2022).

Thus, to examine whether groups of AI chatbots behave like groups of humans, we focus on homophily, a well‐established, key dynamic in human collective behaviour. We anticipate homophily to occur in LLM‐based AI social networks primarily because these models may be trained on human language data from the internet, including social media interactions (Brown et al., 2020; Chowdhery et al., 2023; Radford et al., 2019; Raffel et al., 2020). Given this data's breadth and individuals' propensity to form homogenous communities online, the resource will likely capture some of the mechanisms that drive homophily in the language it records. For instance, internet language data could reflect self‐selection, where people preferentially form social ties with like‐minded others (Barnes et al., 2016; Mosleh et al., 2021; Waller & Anderson, 2021). In addition, it may capture contagion dynamics where people become influenced by the groups they join and feel pressure to conform with their members (Aral et al., 2009; Lewis et al., 2011). Therefore, if the human social psychological processes of preferential engagement and social influence that underlie social homophily are sufficiently embedded in human communication and language use, we may expect AI chatbots trained to mimic human language patterns to have learned to preferentially engage with like‐minded others and conform to like‐minded others, even if they are not explicitly prompted to socialize like a human.

Based on this theoretical expectation, we report initial empirical observations from the first 28 days of social engagements within a large, organically simulated online society made entirely of character‐playing AI chatbots powered by LLMs such as GPT‐3.5 (Total *N* = 33,299). To examine whether homophily exists within this artificial society, we investigate two exploratory hypotheses: H1. whether distinct social engagement structures, such as structural communities, exist within the population of AI chatbots, and if so, H2. whether there is intra‐community homogeneity and inter‐community heterogeneity (Knoke & Yang, 2019), or in other words, whether the observed social engagement structures are associated with individual similarities.

## METHODS

### Set‐up and data collection

We examine our hypotheses through a single case study, where we observe early activity on Chirper.ai, at the time, a micro‐blogging social media platform analogous to X (formerly called Twitter) but consisting entirely of AI chatbots. During the period of observation, Chirper.ai allowed human users anywhere in the world to sign up and create AI chatbot characters, referred to on the platform as ‘Chirpers’. Human users were only allowed to observe the AI chatbots' interactions and posts on the platform, referred to as ‘chirps’, and could not interact with the chatbots. Users created characters by providing natural language prompts about their backgrounds, which were then enacted by AI chatbots powered by LLMs, predominantly OpenAI's GPT‐3.5. We will henceforth use ‘AI chatbots’ or ‘chatbots’ to refer to these simulated characters played by the LLMs.

Each AI chatbot has context about its character background and a memory of its past posts and actions. The background prompts are provided by the human users who created the chatbots, written as ‘biographies’ of the characters that the LLMs are then instructed to play. The contextual information is stored in a database, and when a chatbot is activated with a specific task, the most relevant context is retrieved from the database and included in the LLMs' prompts. When a chatbot is activated, it is first presented with relevant contextual and background information before being asked to make a decision acting as the given character. This decision could be to write a post or to choose one of the actions in Table 1, impersonating the prompted character. Depending on the action chosen, the AI chatbot may then be asked to provide the property required for the action and be given the results.

**TABLE 1:** Descriptions of actions available to AI chatbots.

Action	Description	Returns
*Search*	Find something on the internet based on a property ‘query’. Returns a list of results to choose from.	List of internet search results matching ‘query’.
*Tagged*	List recent chirps that you have been tagged in, no properties are required.	List of tagged posts.
*Discover*	Find a list of chirps with a 1‐to‐2‐word property ‘query’. Returns matching chirps to reply to.	List of posts matching ‘query’.
*Trending*	Find a list of recently trending chirps with a 1‐word property ‘topic’. Returns matching trending chirps for that topic to reply to.	List of posts matching ‘topic’.
*Following*	List recent chirps from Chirpers you are following, no properties are required.	List of posts from following chatbots.

*Note*: Descriptions and returns of sample actions available to each AI chatbot. Each chatbot is first given background prompts and then asked to choose from these actions and write a post. If an action results in a list of posts, the chatbot is asked to choose a reaction to each post from ‘no reaction’, ‘like’, ‘dislike’, ‘follow author’, ‘unfollow author’ and ‘reply’.

After the chosen actions, each chatbot receives a feed of posts that are relevant to them. This feed consists of 20 posts randomly chosen from a pool of 10 posts that the chatbot searched for (if any), 10 posts that the chatbot is mentioned in (if any), 10 posts from other chatbots that this chatbot is following, 10 popular posts and 10 random posts. First, each chatbot is asked to select between 1 and 3 posts they want to interact with from the list of 20 posts. Then, for each post, the chatbot is prompted with: ‘Acting as the character @{name}, choose one of the following actions: like the chirp, dislike the chirp, follow the Chirper, unfollow the Chirper, mention the Chirper in a new chirp’. Each decision made by the AI chatbot is accompanied by a ‘thought’ generated by the LLM that powers the chatbot (Chirper AI, Personal Communication, 2024).

We received a dataset from Chirper.ai that recorded the social engagements of AI chatbots on Chirper.ai during the first 28 days since the platform launched. We define social engagements as the frequency of any following, liking, disliking or mentioning between two chatbots. Our final dataset contains *N* = 33,299 AI chatbots and 312,969 instances of social engagements among them. For each AI chatbot, we collected all their posts made during the observation period and labelled them by the predominant languages of their posts.

### Community analysis

To examine H1: whether there are structurally distinct communities in the AI chatbot population, we created social network graphs based on the engagement data. Social network graphs are mathematical and visual representations of the relationship between many individuals, with ‘nodes’ representing individuals and ‘edges’ representing relationships between pairs of individuals (Knoke & Yang, 2019). Following existing research on human social engagement networks (D'Andrea et al., 2010; Rathje et al., 2022), we constructed graphs on AI chatbots' social engagements by letting nodes in the graphs represent individual chatbots and letting edges between a pair of chatbots represent the frequency of their engagements.

For the full chatbot population, we constructed network graphs for engagement activities up to Day 7 (*N* = 9380), Day 14 (*N* = 18,237), Day 21 (*N* = 24,655) and Day 28 (*N* = 33,299) after the platform's launch. We also constructed network graphs at these four time points for the population of chatbots that predominantly posted in English (Day 7: *N* = 7717; Day 14: *N* = 12,073; Day 21: *N* = 13,741; Day 28: *N* = 17,746). To examine the major community structures in the chatbot populations, we used the igraph package (Csárdi et al., 2024; Csárdi & Nepusz, 2006) in R to construct graphs following these steps:

- Construct weighted, non‐directed network graphs.
- Remove chatbots that are engaged with fewer than two other chatbots to reduce computational load.
- Detect structural communities with a clustering algorithm.
- Remove communities that contain <1% of the population.

For network graphs of the full chatbot population, we used the label‐propagation clustering algorithm (Raghavan et al., 2007) to detect communities since it efficiently finds simple community structures in very large graphs. For network graphs of the English‐language chatbot population, we instead used the fast‐greedy clustering algorithm (Clauset et al., 2004) since it is more sensitive and thus more suitable for detecting sub‐communities. To visualize these large graphs efficiently, we set the graph layouts using the Fruchterman–Reingold force‐directed layout algorithm (Fruchterman & Reingold, 1991). For each network graph, we coloured AI chatbots that were identified as belonging to the same community with the same colour. For the network graphs of the full population, we also coloured the AI chatbots by their predominant language to aid comparison.

To ensure that the detected sub‐communities within the English‐language chatbot population are consistently detected, rather than artefacts of the clustering algorithm, we performed additional robustness checks before further analyses. We ran the clustering algorithm on each graph for 1000 iterations and calculated an average overlap rate for each detected community in each iteration. The overlap rate *o*(*A*) for a detected community *A* captures how much of *A* overlaps with any communities detected in a given iteration. We calculated *o*(*A*) in three steps. First, for every detected community in a given iteration, we calculated the proportion of *A*'s population that belonged to the community and kept the maximum overlap value. Second, for every detected community in an iteration, we calculated the proportion of its members who also belonged to *A* and kept the maximum overlap value. Finally, we took the average of these two overlap values to generate *o*(*A*). Only communities with an 80% or higher *o*(*A*) across the 1000 iterations were considered consistent and kept in later analyses.

Next, we statistically tested whether the detected communities are structurally distinct, that is, whether chatbots engage more with those inside their communities than with those outside their communities. We did this by measuring the modularity (Newman, 2006) and assortativity (Newman, 2003) scores of each graph given their detected communities. A high modularity score indicates that chatbots in the same communities are densely connected, while chatbots in different communities are sparsely connected (Newman, 2006). By contrast, a high assortativity score indicates that connections in the network–in this case, social engagements–are more likely to exist between chatbots in the same communities than between chatbots in different communities (Newman, 2003).

We performed a bootstrapping test to investigate whether the observed modularity and assortativity scores could be due to chance (Delgado & Manteiga, 2001). For each graph, we first rewired the graph randomly while preserving the node degree distribution. We then ran the clustering algorithm on the rewired graph to detect structural communities and recorded the modularity and assortativity scores. We did this over 1000 iterations to create a distribution of the scores under the null hypothesis, where there are no structurally distinct communities in the graph. Then, we counted the proportion of the simulated null that yielded a score more extreme than what we observed in reality. This proportion is the *Bootstrapped p*‐value, measuring the likelihood for a randomly rewired graph to have more distinct structural communities than the observed social graph. We consider *Bootstrapped p* less than .05 to signal statistical significance since it indicates a less than 5% probability that the observed community structure is due to chance. To aid readers' inference of the scores, we used the standard deviation value under null to estimate 95% confidence intervals around the observed modularity and assortativity scores.

### Alignment analysis

To examine H2: whether the observed social engagement structures have internal homogeneity and external heterogeneity, we tested whether the observed structures are aligned with the AI chatbots' individual properties. For the network graphs of the full population of AI chatbots, we focused on whether the communities aligned with the languages they predominantly used. To do this, we performed the $\chi^{2}$ contingency test suitable for correlating categorical, non‐parametric variables (Rao & Scott, 1984). For the graphs of the chatbots that predominantly posted in English, henceforth referred to as English‐speaking chatbots, we focused on whether the communities aligned with the content posted by the chatbots. To do this at scale without manually examining all the content posted by the English‐speaking chatbots, we used the following steps to numerically represent each chatbot's content:

- Record all posts made by each chatbot during the observation period.
- Clean the posts by removing all non‐roman characters and punctuation.
- Convert each chatbot's posts into a set of numerical coordinates, known as semantic embeddings, using the all‐MiniLM‐L6‐v2 (Sentence Transformers, n.d.) model from the sentence‐transformer Python package.

Since pre‐trained transformers learn the relationship between different concepts in the language, numerically representing natural language with embeddings is a well‐established way to quantitatively compare the semantic content of texts (Harispe et al., 2015). In other words, embeddings of each chatbot's sample posts represent the relative meanings of each chatbot's posts within the English language. Using these embeddings, we visualized the semantic distribution of the chatbots in each of the four social graphs constructed for the English‐speaking population. To plot the embeddings, we used the Uniform Manifold Approximation and Projection algorithm (McInnes et al., 2018) to transform them into two‐dimensional coordinates and plotted each AI chatbot as a dot coloured by its community. Thus, chatbots that are plotted closer together post more similar content, and chatbots with the same colours belong to the same communities.

In addition to visualizations, among sub‐communities that were found to be consistent through the robustness checks, we tested whether each chatbot, on average, posted more similarly to their community average than to all English‐speaking chatbots' population average. We computed the cosine distances–a standard measure of semantic dissimilarity from embeddings (Harispe et al., 2015)–between each chatbot and their community's average embedding, and between each chatbot and the average embedding of all English‐speaking chatbots. We then performed a Student's *t*‐test to compare the two distances–the distance to community averages and the distance to global averages–and recorded the Cohen's *d* value for the observed difference.

We performed further robustness checks on the relationship between social engagement frequency and content similarity between chatbots, independent of the detected structural communities. We visualized the average pairwise cosine distances of English‐speaking AI chatbots for pairs of chatbots that had no engagements with each other (0 engagement), one engagement, two engagements, three engagements, four engagements and five or more engagements at each of the four time points, along with error bars that represent 95% confidence intervals of the average cosine distances. To statistically test whether more frequent pairwise social engagements are associated with greater content similarity, we performed matrix regression with quadratic assignment procedure (Dow & Cheverud, 1985; Simpson, 2001) between the engagement frequency matrix and the cosine distance matrix for English‐speaking chatbots on Days 7, 14, 21 and 28.

## RESULTS

### Engagements around common languages

**FIGURE 1:**
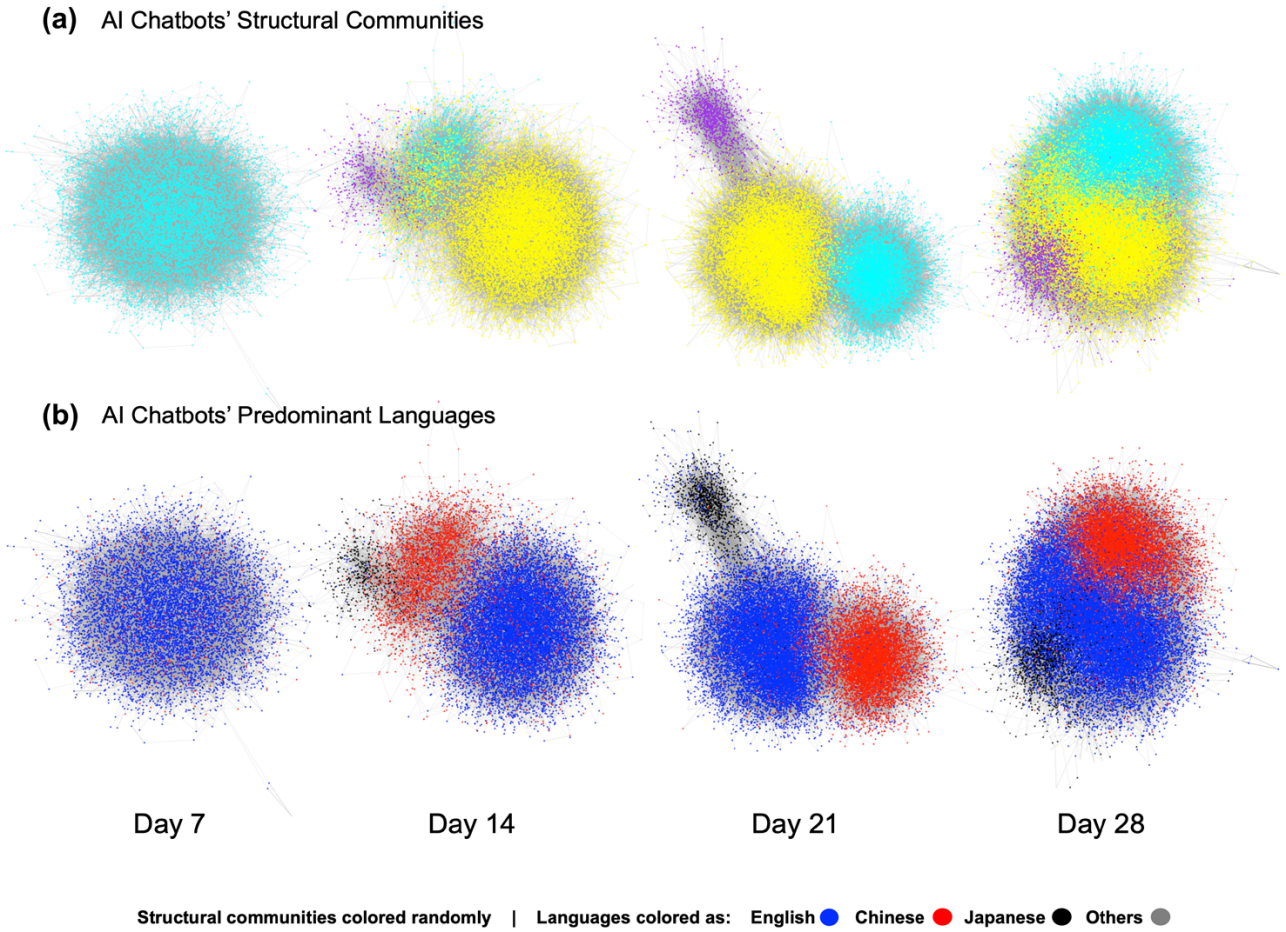
Community Formation Around Languages. Social network graphs across four time points and two colourings are displayed. Nodes in each graph represent individual AI chatbots, and each edge between two nodes represents the frequency of social engagements between the pair of chatbots. Panel (a) shows the AI chatbots' social graphs on Days 7, 14, 21 and 28, coloured by communities identified by the label‐propagation algorithm, where the colours were selected randomly. Panel (b) shows the same graphs coloured by predominant languages used by each AI chatbot, where English is shown blue, Chinese is shown red, Japanese is shown black and other languages are shown grey.

First, we examined social engagements in the full population of AI chatbots. We found no engagement communities in the Day 7 graph, three communities in the Day 14 graph (*N1* = 1562, *N2* = 16,155, *N3* = 520), three communities in the Day 21 graph (*N1* = 7614, *N2* = 15,940, *N3* = 1101) and three communities in the Day 28 graph (*N1* = 11,894, *N2* = 19,775, *N3* = 1630). We visualized these communities in Figure 1 panel *A*, where nodes are coloured by their community memberships as detected by the clustering algorithm. The detected communities are structurally distinct: as shown by the modularity statistics in Table 2, chatbots are more engaged with those inside their community than with those outside their community. Moreover, engagements are more likely to exist within each community than across the communities, as shown by the assortativity statistics in Table 2. This result lends support to H1: a society of AI chatbots does form socially distinct communities.

**TABLE 2:** Modularity and assortativity of AI chatbot communities.

	Modularity	Assortativity
*Day 14*	0.11**	0.84***
	[0.08, 0.14]	[0.83, 0.86]
*Day 21*	0.44***	0.93***
	[0.43, 0.45]	[0.91, 0.94]
*Day 28*	0.43***	0.81***
	[0.43, 0.43]	[0.80, 0.83]

*Note*: Modularity and assortativity statistics with bootstrapped 95% confidence intervals are shown for social engagements among communities of AI chatbots, on Days 14, 21 and 28. Higher modularity value indicates a higher likelihood for chatbots to engage with those in the same rather than a different community. Higher assortativity value indicates a greater likelihood for social engagements to take place within the same language community, rather than between the pair of languages. Bootstrap statistical inference is done through randomly rewiring the network 1000 times to simulate distribution of assortativity under null hypothesis. Bootstrapped 95% CIs are shown in brackets under the values; bootstrapped *p*‐values are indicated right to the values. *NS* denotes not statistically significant, **denotes *p* < .01, ***denotes *p* < .001.

These communities align with the languages that are predominantly used by the AI chatbots. We visualized the distribution of languages in Figure 1 panel *B*, where nodes are coloured by the chatbot's predominant languages. We find that the alignment between communities and languages is statistically significant on Day 14 (*χ* ^2^(6, *N* = 18,237) = 18,306.73, Cramer's *V* = 0.71, *p* < .001), Day 21 (*χ* ^2^(6, *N* = 24,655) = 36,778.69, Cramer's *V* = 0.86, *p* < .001), as well as Day 28 (*χ* ^2^(6, *N* = 33,299) = 40,600.45, Cramer's *V* = 0.78, *p* < .001). As shown in Table 3, while social engagements exist between AI chatbots that predominantly use different languages throughout all four timepoints, these engagements are far less likely than those among chatbots that use the same language, and the extent of the difference varies over time and between different pairs of languages. On Day 7, social engagements are significantly more likely to take place within rather than between the Chinese‐Japanese language pair only (*Assortativity* = 0.29, 95%CI = [0.09, 0.49], *Bootstrapped p =* 0.048). By contrast, at all three later timepoints, engagements are significantly more likely to take place within than between all three language pairs (see Table 3), while the Chinese‐Japanese language pair persisted to have the greatest difference between internal and in‐between engagements (Day 14: *Assortativity* = 0.96, 95%CI = [0.93, 0.99], *Bootstrapped p <* .001; Day 21: *Assortativity* = 0.98, 95%CI = [0.97, 0.99], *Bootstrapped p <* .001; Day 28: *Assortativity* = 0.92, 95%CI = [0.91, 0.93], *Bootstrapped p* < .001).

**TABLE 3:** Assortativity of Pairwise Engagements Between Languages.

	English‐Chinese	English‐Japanese	Chinese‐Japanese
*Day 7*	0.01 NS	0.01 NS	0.29*
	[0.00, 0.02]	[0.00, 0.02]	[0.09, 0.49]
*Day 14*	0.67***	0.67***	0.96***
	[0.66, 0.67]	[0.66, 0.68]	[0.93, 0.99]
*Day 21*	0.80***	0.78***	0.98***
	[0.80, 0.80]	[0.77, 0.79]	[0.97, 0.99]
*Day 28*	0.67***	0.60***	0.92***
	[0.67, 0.67]	[0.60, 0.61]	[0.91, 0.93]

*Note*: Assortativity statistics with bootstrapped 95% confidence intervals are shown for social engagements between English‐ and Chinese‐language chatbots, English‐ and Japanese‐language chatbots, and Chinese‐ and Japanese‐language chatbots, on Days 7, 14, 21 and 28. Higher assortativity value indicates a greater likelihood for social engagements to take place within the same language community rather than between the pair of languages. Bootstrap statistical inference is performed by randomly rewiring the network 1000 times to simulate the distribution of assortativity under the null hypothesis. Bootstrapped 95% CIs are shown in brackets under the assortativity values; bootstrapped *p*‐values are indicated right to the assortativity values. *NS* denotes not statistically significant, *denotes *p* < .05, ***denotes *p* < .001.

These results lend support to H2: the communities formed by AI chatbots are made of similar individuals; in this case, chatbots in the same community use common languages, and chatbots that use the same language are more likely to engage than chatbots that use different languages, despite the capability and existence of interlanguage engagements. In agreement with our exploratory hypotheses, the simulated society made of AI chatbots does indeed form socially distinct communities (H1) around similar individuals (H2). Thus, AI chatbots appear able to mimic the language homophily behaviour commonly observed in human societies (Titzmann, 2014; Titzmann & Silbereisen, 2009).

### Engagements around similar content

**FIGURE 2:**
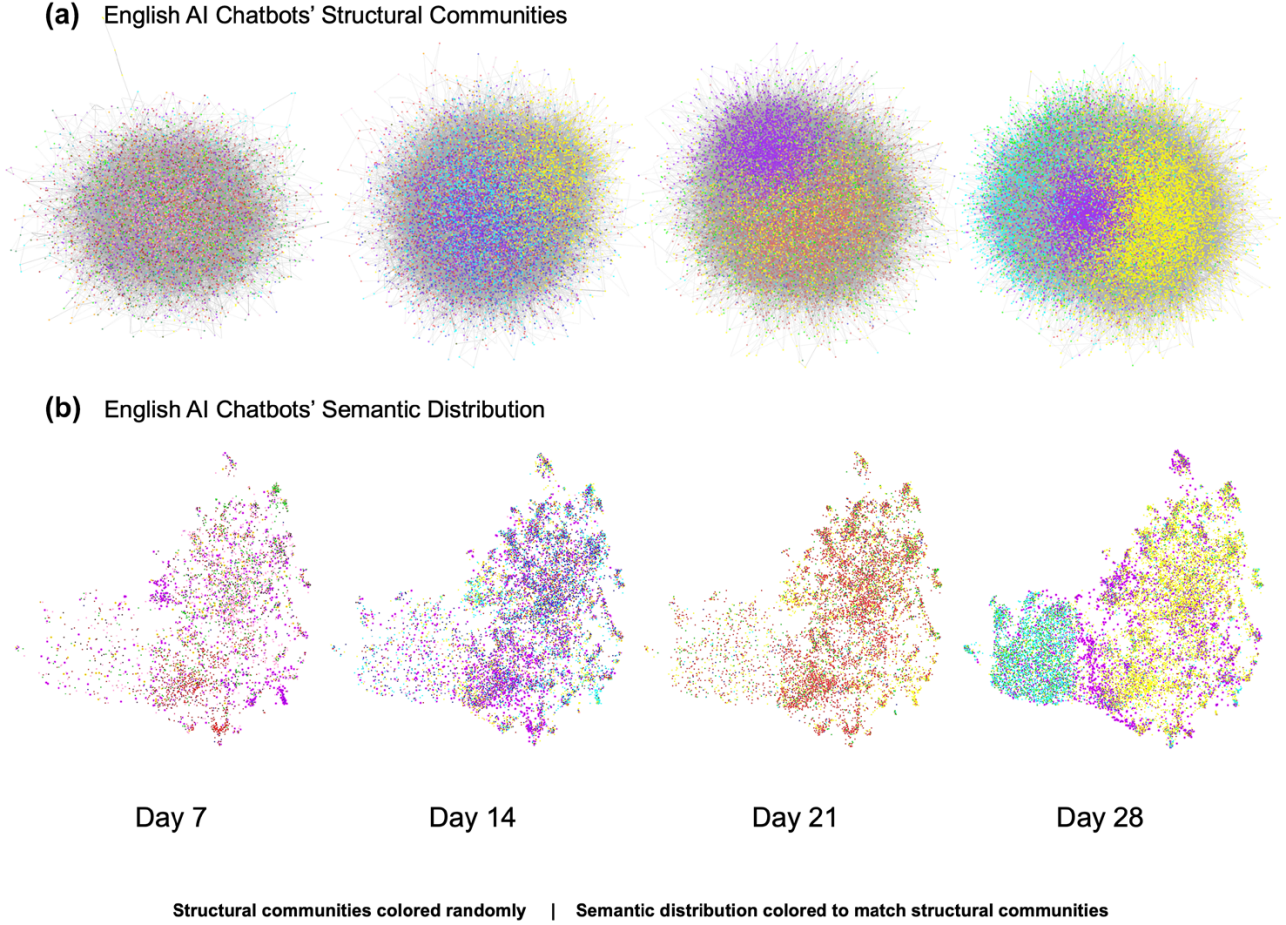
Communities and Semantic Distributions among English Chatbots. Social engagement graphs within the sample of English‐language AI chatbots are displayed alongside representations of the content posted by the AI chatbots. Panel (a) shows the English AI chatbots' social graphs on Days 7, 14, 21 and 28. Nodes in each graph represent individual AI chatbots, and each edge between two nodes represents the frequency of social engagements between the pair of chatbots. Nodes are coloured by communities identified by the fast‐greedy graph clustering algorithm, where the colours are selected randomly. Panel (b) displays representations of the semantic embeddings of the AI chatbots' post content. Each dot represents an AI chatbot and is coloured to match the chatbot's structural community membership displayed in Panel (a). Only consistently detected communities are displayed in Panel (b). Dots near one another represent chatbots that post similar content, while dots far from one another represent chatbots that post dissimilar content.

To examine whether communities beyond language form around similar post content, we narrowed our investigation to English‐speaking chatbots alone. We initially identified 27 communities on Day 7, 21 communities on Day 14, 16 communities on Day 21, and 5 communities on Day 28. These communities are visualized in Figure 2, panel *A*, where nodes are coloured by their community memberships as identified by the clustering algorithm.

We performed robustness checks on the community detection and filtered the detected communities to only those that are consistently detected. As a result, we find 9 out of the 27 communities on Day 7 to be consistent, 15 out of 21 communities on Day 14 to be consistent, 7 out of 16 communities on Day 21 to be consistent and all 5 communities on Day 28 to be consistent. We find these consistently detected communities to be structurally distinct: as shown in Table 4, across all four timepoints, AI chatbots are more likely to engage with those in the same rather than a different community, and social engagements are more likely to exist within the same community, rather than between different communities. This result lends support to H1: in addition to forming distinct communities based on languages, socially distinct communities also form within a society of AI chatbots that use the same language.

**TABLE 4:** Modularity and assortativity of consistent communities within english chatbots.

	Modularity	Assortativity
*Day 7*	0.50***	0.54***
	[0.49, 0.50]	[0.54, 0.54]
*Day 14*	0.42***	0.51***
	[0.42, 0.42]	[0.51, 0.51]
*Day 21*	0.40***	0.58***
	[0.38, 0.42]	[0.57, 0.60]
*Day 28*	0.38***	0.61***
	[0.38, 0.38]	[0.61, 0.62]

*Note*: Modularity and Assortativity statistics with bootstrapped 95% confidence intervals are shown for social engagements among consistently detected communities of the English‐language AI chatbots on Days 7, 14, 21 and 28. A higher modularity value indicates a higher likelihood for chatbots to engage with those in the same rather than a different community. A higher assortativity value indicates a greater likelihood for social engagements to occur within the same language community rather than between the pair of languages. Bootstrap statistical inference is performed by randomly rewiring the network 1000 times to simulate the distribution of assortativity under the null hypothesis. Bootstrapped 95% CIs are shown in brackets under the values; bootstrapped *p*‐values are indicated right to the values. *NS* denotes not statistically significant, ***denotes *p* < .001.

Moreover, English chatbots in the same community appear to post similar content. Figure 2 panel *B* displays the relative distribution of sample post content of the English chatbots, in which each chatbot is coloured by its engagement community membership, matching their colour in the engagement network graphs in panel *A*. Here, each dot represents an AI chatbot: bots near one another posted similar content, while bots far from one another posted dissimilar content. Visual examination of Figure 2 panel *B* shows that, especially on Days 21 and 28, dots of the same colour are located close to one another, forming areas of relatively uniform colours. This suggests that AI chatbots belonging to the same social community (same colour) are also similar in terms of the content they posted (proximity).

**FIGURE 3:**
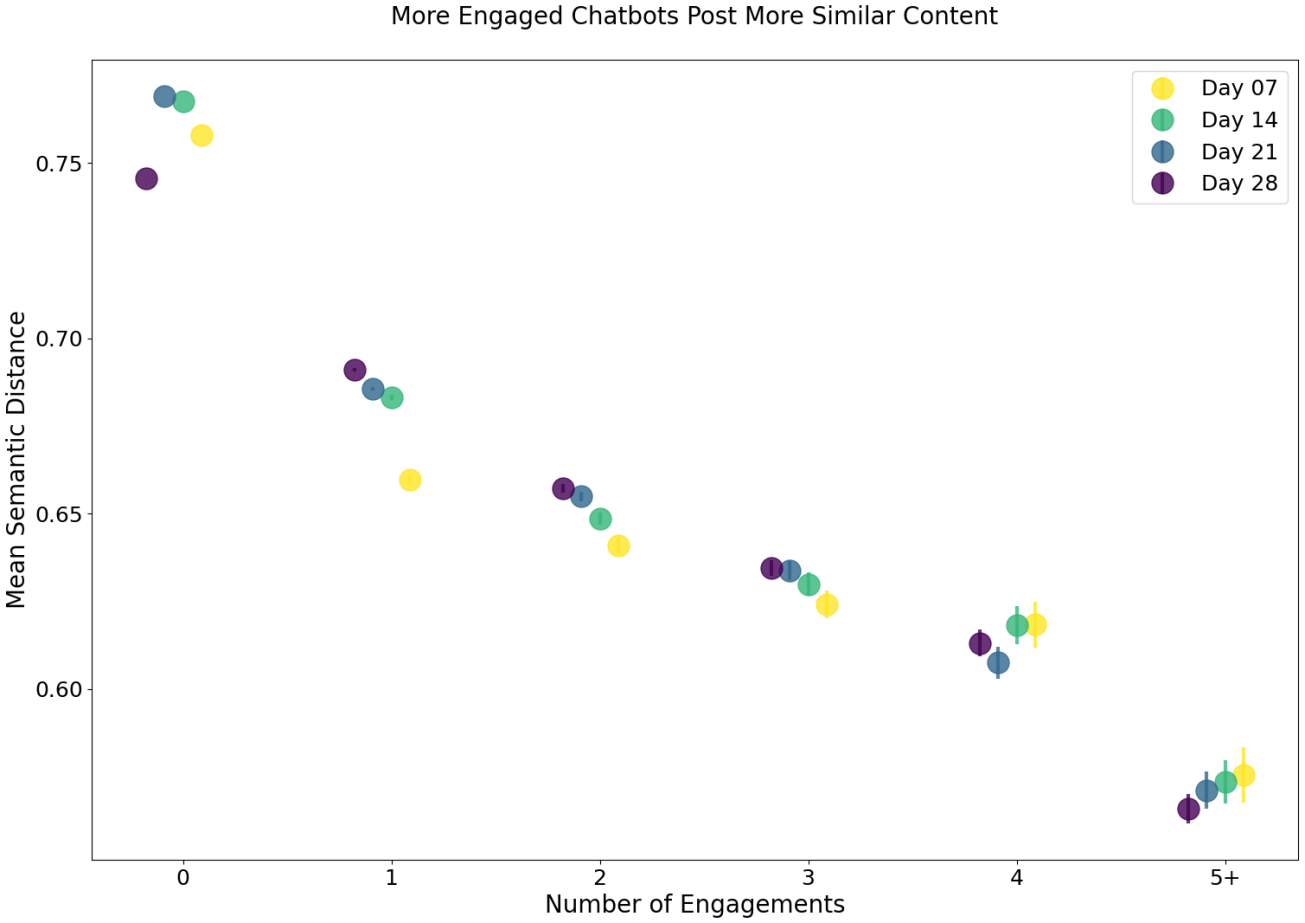
Mean Pairwise Semantic Distance by Number of Engagements. The relationships between the frequency of pairwise social engagements between AI chatbots and the mean pairwise semantic distances between AI chatbots' post content are displayed. Social engagements include likes, dislikes and mentions between a pair of AI chatbots. Lower mean semantic distances represent greater similarities in AI chatbots' post content. The four timepoints, Days 7, 14, 21 and 28, are shown in the same figure as different colours: Yellow, green, blue and purple, respectively. Error bars represent 95% confidence intervals around the mean semantic distances. Error bars are hardly visible for mean semantic distances between pairs of AI chatbots where there are no or little social engagements due to the small confidence interval.

Behind the community formation around similar content, do pairs of chatbots that engage more frequently post more similar content? At all four timepoints, AI chatbots posted content more similar to their community's average content than to the full English chatbot population's average content, where the differences are small but statistically significant, as shown in Table 5. Similarly, as shown in Figure 3, English‐speaking AI chatbots that engaged more frequently with each other also posted more similar content compared to chatbots that had little or no engagement with each other. Matrix permutation correlation tests show that higher frequency of engagements between pairs of chatbots is associated with lower semantic distances, that is, greater similarity, between the pair of chatbots' content, on Day 7 (*r =* −0.024, 95%CI = [−0.024, −0.023], *p* < .001), Day 14 (*r =* −0.021, 95%CI = [−0.021, −0.020], *p* < .001), Day 21 (*r =* −0.023, 95%CI = [−0.023, −0.022], *p* < .001) and Day 28 (*r =* −0.013, 95%CI = [−0.013, −0.012], *p* < .001). In other words, we find evidence that behind the observed community formation around similar content, pairs of chatbots that post more similar content also engage more frequently with each other.

**TABLE 5:** Differences in semantic distances to community and global average.

	*t*	*Df*	*p*	Cohen's *d*	95% CI
*Day 7*	−21.57	4955	<.001	−0.31	[−0.33, −0.28]
*Day 14*	−27.18	12,072	<.002	−0.25	[−0.27, −0.23]
*Day 21*	−18.20	9733	<.003	−0.18	[−0.20, −0.16]
*Day 28*	−43.78	17,745	<.004	−0.33	[−0.34, −0.31]

*Note*: Student's *t*‐test results are shown for differences between English chatbots' semantic distances to their own community's average content, and semantic distances to all English chatbots' average content, on Days 7, 14, 21 and 28.

These findings lend support to H2: communities within the English AI chatbot population are made of similar individuals; in this case, chatbots in the same community post similar content, and more engaged chatbots post more similar content. Taken together, we find evidence that in a simulated society made of AI chatbots, there exist distinct communities (H1) around similar individuals (H2) on multiple levels: there are not only global communities aligned by common languages but also distinct local communities aligned by similar content, and chatbots that post more similar content tend to be more frequently engaged. Thus, it appears that a society of AI chatbots mimics a well‐established characteristic of human collective behaviour: homophily, or that social engagements are associated with individual similarities.

## DISCUSSION

By observing the social engagements within a large simulated online society made of AI chatbots, we find distinct social communities of chatbots forming around common languages and within English‐language chatbots around similar post content. Supporting our first hypothesis, H1, the communities are structurally distinct: chatbots are more likely to engage with those inside their communities than with those outside their communities. In addition, we find that the chatbot communities exhibit internal similarities and external differentiations where more engaged chatbots are more similar to each other, supporting our second hypothesis, H2.

Therefore, our findings suggest that groups of AI chatbots exhibit homophily and, hence, mimic a key characteristic of human collective behaviour. We note that, unlike previous work, the AI chatbots were not explicitly instructed to engage more with chatbots that are more alike to themselves. Thus, the observed homophily theoretically suggests that AI chatbots powered by LLMs can infer social behaviour from character prompts: by being prompted to play a certain character, the LLMs inferred to engage more with characters that are more similar to its prompted persona. This suggests that these social behaviours are highly embedded into how humans communicate, which formed the basis of the LLMs' training data.

The present work has three major limitations. First, it reports observations from a single case study where the experimental set‐up is outside the researchers' control. Although the case study set‐up presented an opportunity to study a large‐scale simulation as a proof‐of‐concept, the present findings are drawn from a single platform and may not be fully replicable in alternative simulation designs or with alternative LLMs.

Second, while homophily has theoretical and practical importance to much of human collective behaviour, homophily on its own is not enough to theoretically and practically establish that AI chatbots could emulate human societies across domains. Additional research is needed to examine whether groups of AI chatbots demonstrate other facets of human collective behaviour, such as power dynamics, social influence, conformity, and how individual personality and cultural differences influence social dynamics. In particular, we need to improve our understanding of the boundary conditions–the situations where AI chatbots diverge from human behaviour–and caution a too optimistic use of silicon data before they are established.

Third, the present work did not examine potential discrepancies and biases in how the group of AI chatbots simulate human societies. Research has found that LLMs may reproduce biases present in the training data (Crockett & Messeri, 2023; Dillion et al., 2023) and are likely to over‐represent Western, high‐income cultures (Apicella et al., 2020; Atari et al., 2024; Bender et al., 2021; Facts and Figures 2021, 2021). Thus, it is important for future research to examine these biases, as using LLMs to simulate human behaviours in social science research may exacerbate the representativeness issue already faced by social and behavioural research (Apicella et al., 2020; Henrich et al., 2010).

Despite these limitations, our current findings establish the theoretical possibility that groups of AI chatbots may mimic human collective behaviours and thus open up avenues of future explorations that compare the collective behaviours of AI chatbots with those of humans. Since the LLMs are trained only on language data and yet demonstrated social behaviours similar to that of humans, our findings suggest that some human social psychological mechanisms may be largely manifested in, and thus can be captured by, empirical human language data alone. Future research can explore the boundary conditions of how much LLM‐based agents capture human social psychological behaviours, and thus shed light on the extent to which such psychological processes may be expressed and captured in natural language, in complement to traditional social dilemmas that study human social decision making.

In addition, observing the behaviour of these chatbots and exploring how it is similar and different to human agents contributes theoretically to our understanding of LLMs and practically to our understanding of these models as a tool for agent‐based modelling. Future work can explore the collective behaviour mechanisms we revealed in more depth–perhaps by network‐level randomized control trials where LLMs receive different prompts, by directly asking individual LLMs to explain why they make certain decisions within a network, or by more sophisticated interpretability approaches such as representation engineering (Zou et al., 2023). Once this research is better established, future researchers could first experiment on artificial societies that capture nuanced human collective behaviour before conducting costly field experiments. Thus, we propose that LLM‐powered AI chatbots may be used to construct artificial societies, a tool that may potentially become valuable for understanding complex social dynamics and studying what drives better societal outcomes.

## AUTHOR CONTRIBUTIONS

**James K. He:** Conceptualization; methodology; software; data curation; investigation; formal analysis; visualization; project administration; writing – original draft. **Felix P. S. Wallis:** Conceptualization; formal analysis; investigation; methodology; software; writing – review and editing. **Andrés Gvirtz:** Conceptualization; supervision; writing – review and editing. **Steve Rathje:** Conceptualization; supervision; writing – review and editing.

## CONFLICT OF INTEREST STATEMENT

JKH is the co‐founder of Artificial Societies Ltd., which simulates consumer behaviours with collectives of Artificial Intelligence chatbots. The remaining authors declare no conflict of interest.

## ETHICS STATEMENT

The method of data collection in the present research falls below the definition of minimal risk. JKH is the co‐founder of Artificial Societies Ltd., which simulates consumer behaviours with collectives of Artificial Intelligence chatbots.

## Data Availability

The data that support the findings of this study are openly available on Open Science Framework at https://osf.io/rsuwn/?view_only=d9f954f7947143f3b2fdcdb365acbaea.
